# The inconstancy of the transient climate response parameter under increasing CO_2_

**DOI:** 10.1098/rsta.2014.0417

**Published:** 2015-11-13

**Authors:** J. M. Gregory, T. Andrews, P. Good

**Affiliations:** 1 NCAS-Climate, University of Reading, Reading, UK; 2 Met Office Hadley Centre, FitzRoy Road, Exeter, UK

**Keywords:** climate sensitivity, ocean heat uptake, radiative forcing, climate change, climate modelling

## Abstract

In the Coupled Model Intercomparison Project Phase 5 (CMIP5), the model-mean increase in global mean surface air temperature *T* under the 1pctCO2 scenario (atmospheric CO_2_ increasing at 1% yr^−1^) during the second doubling of CO_2_ is 40% larger than the transient climate response (TCR), i.e. the increase in *T* during the first doubling. We identify four possible contributory effects. First, the surface climate system loses heat less readily into the ocean beneath as the latter warms. The model spread in the thermal coupling between the upper and deep ocean largely explains the model spread in ocean heat uptake efficiency. Second, CO_2_ radiative forcing may rise more rapidly than logarithmically with CO_2_ concentration. Third, the climate feedback parameter may decline as the CO_2_ concentration rises. With CMIP5 data, we cannot distinguish the second and third possibilities. Fourth, the climate feedback parameter declines as time passes or *T* rises; in 1pctCO2, this effect is less important than the others. We find that *T* projected for the end of the twenty-first century correlates more highly with *T* at the time of quadrupled CO_2_ in 1pctCO2 than with the TCR, and we suggest that the TCR may be underestimated from observed climate change.

## 1. Introduction

The transient climate response (TCR) is defined [1] as the global mean surface air temperature change *T* (with respect to the unperturbed state) under the 1pctCO2 scenario, in which the atmospheric CO_2_ concentration increases at 1% yr^−1^, at the time when it reaches twice its initial value (after 70 years, 1.01^70^=2). The TCR is regarded as a benchmark for the magnitude of climate change to be expected from an atmosphere–ocean general circulation model (AOGCM) in response to increasing radiative forcing, it being assumed that many aspects of climate change scale with *T* [2]. The spread of TCR in the AOGCMs of the Coupled Model Intercomparison Project Phase 5 (CMIP5) is 1.2–2.4 K (90% confidence interval) [3].

The 1pctCO2 forcing scenario is of plausible magnitude for the twenty-first century but idealized. To make a link with more realistic scenarios, Gregory & Forster [4] proposed a simple model of global mean climate change, in which *F*(*t*)=*ρT*(*t*), where *F* is the effective radiative forcing (henceforth ‘forcing’ for brevity), *t* is time and *ρ* is the climate resistance (W m^−2^ K^−1^). In this model, *T*(*t*) is instantaneously related to *F*(*t*), and the TCR is *F*_2×_/*ρ*, where *F*_2×_ is the forcing due to doubling the CO_2_ concentration. We call this the ‘zero-layer model’ [5], because finite heat capacity does not appear in it.

In the zero-layer model, we assume that all the heat added to the climate system is stored in the ocean, so that the net downward radiative flux *N* at the top of the atmosphere equals the rate of ocean heat uptake. An observational assessment of heat uptake by the climate system during 1971–2010 indicates that 93% of it is accounted for by increased ocean heat content, with most of the remainder being latent heat associated with loss of land ice [6]. CMIP5 AOGCMs do not simulate most changes in the mass of land ice. Consequently, in these models, *N* is a very good approximation to the rate of ocean heat uptake, for time scales longer than about a year (fig. 13.8 of [7,8]), while on shorter time scales the heat capacity of land and atmosphere cannot be neglected.

We can write *ρ*=*α*+*κ*, where *α* is the climate feedback parameter and *κ* is the ocean heat uptake efficiency [9,10]. These quantities, respectively, measure the tendency of the surface climate system, as it warms up, to lose more heat to space (by net outgoing radiation, at a rate *R*=*αT*) and into the ocean (at a rate *N*=*κT*). Thus *F*=*R*+*N*. The temperature *T* is a skin temperature, a property of the surface climate system, including a shallow upper-ocean layer, which is regarded as having negligible heat capacity. The deep ocean is regarded as a heat sink with infinite heat capacity—it absorbs heat but does not warm up.

The zero-layer model predicts *T*=*F*/*ρ* for time-dependent forcing. This is formally analogous to the prediction of the steady-state *T*=*F*/*α* for constant forcing. The equilibrium climate sensitivity (ECS), defined as *T*(=*F*_2×_/*α*) for constant 2×CO_2_ concentration, is a benchmark originally introduced for climate models with ‘slab’ or ‘mixed-layer’ oceans, which cannot be used for projecting time-dependent climate change, because they do not model ocean heat uptake realistically. The ocean heat uptake efficiency does not affect ECS because there is no ocean heat uptake at equilibrium, and the forcing is balanced only by increased radiation to space. Since *ρ*=*α*+*κ*>*α*, TCR is less than ECS, by about one-third for the 1pctCO2 scenario in CMIP5 [11], because ocean heat uptake mitigates the rate of warming.

We recommend the term ‘transient climate response parameter’ (TCRP) for *T*/*F*=1/*ρ* (K W^−1^ m^2^), the increase in global mean surface air temperature per unit increase in forcing (in any scenario, not necessarily 1pctCO2). We use ‘parameter’ in naming the TCRP to denote the same relationship to the TCR as exists between the climate sensitivity parameter 1/*α* and the ECS, the corresponding quantities for equilibrium climate change. Held *et al.* [12] propose ‘transient climate sensitivity’ for 1/*ρ*, but Merlis *et al.* [13] use that phrase for *F*_2×_/*ρ*, and this could be confusing; moreover, the mention of ‘sensitivity’ could cause confusion with ECS.

The zero-layer model is a helpful interpretative picture, but it is accurate only if the TCRP 1/*ρ* is constant. Gregory & Forster [4] showed that the TCRP is nearly constant for the HadCM3 AOGCM under anthropogenic historical and Special Report on Emission Scenarios (SRES) forcing, and for the AOGCMs of the Coupled Model Intercomparison Project Phase 3 (CMIP3) under 1pctCO2 up to 2×CO_2_, but that it cannot be constant in scenarios where the forcing stabilizes, to which the ECS eventually applies. Gregory & Forster [4] also noted that, in the 1pctCO2 experiments, the increase in *T* for the second doubling of CO_2_, from 2×CO_2_ to 4×CO_2_, is greater than the TCR, i.e. *T* under the first doubling. The forcing increase on doubling CO_2_ from any level is always *F*_2×_, if we make the usual assumption (to which we return in §5) that CO_2_ forcing depends logarithmically on CO_2_ concentration *C* according to 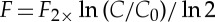 [14–16], where *C*_0_ is a reference level. Hence, the larger *T* increase for the second doubling indicates that the TCRP increases (*ρ* decreases) as the experiment proceeds. This behaviour is shown by both CMIP3 and CMIP5 AOGCMs.

The aim of this paper is to explain the inconstancy of TCRP in CMIP5 AOGCMs under CO_2_ forcing, which has implications for their projections under representative concentration pathway (RCP) scenarios. This leads us to review several of the issues investigated by Long & Collins [17] from a different perspective. We use data from CO_2_ and RCP experiments with the AOGCMs listed in table 1, and to remove model climate drift we subtract the state of the parallel pre-industrial control run (piControl, with constant atmospheric composition), as a function of time. We begin by describing two simple models which are helpful in interpretation of the AOGCM results.

**Table 1.** Parameters of the 16 CMIP5 AOGCMs used in this study. The last two lines of the table show ensemble mean and standard deviation. The TCR is the transient climate response evaluated as the time-mean *T* with respect to control for years 61–80 in the 1pctCO2 scenario, and *T*_140_ is for year 140 in the same scenario, estimated from a linear regression against time for years 121–140. The parameters *α*, *κ* and *ρ* for years 61–80 and 121–140 of 1pctCO2 are evaluated as the ratios of the time-means (*F*−*N*)/*T*, *N*/*T*, *F*/*T*, using the logarithmic formula for *F*(CO_2_) with the 2×CO_2_ forcing *F*_2×_ obtained by regression of *N* against *T* in years 1–20 of the abrupt4xCO2 scenario. ECS is the effective climate sensitivity estimated as *F*_2×_/*α*, using the TCR *α*. For RCP4.5 and RCP8.5, *N* and *T* are the time-means with respect to control for years 2081–2100, and *κ*=*N*/*T*. TCR, *T*_140_, RCP *T* and ECS are in K, *F*_2×_ in W m^−2^, *α*, *κ* and *ρ* in W m^−2^ K^−1^.

					1pctCO2 years 61–80			1pctCO2 years 121–140			RCP8.5 2081–2100			RCP4.5 2081–2100			
AOGCM	*F*_2×_	TCR	*T*_140_	*T*_140_ ÷ TCR	*α*	*κ*	*ρ*	*α*	*κ*	*ρ*	*N*	*T*	*κ*	*N*	*T*	*κ*	ECS
ACCESS1.0	3.44	1.98	4.59	2.32	1.00	0.74	1.74	0.99	0.51	1.51	2.56	4.46	0.57	1.37	2.63	0.52	3.45
ACCESS1.3	3.42	1.65	4.02	2.43	1.22	0.85	2.07	1.14	0.62	1.76	2.65	4.25	0.62	1.33	2.37	0.56	2.80
CanESM2	4.11	2.41	5.24	2.17	1.14	0.56	1.70	1.14	0.45	1.59	2.51	5.31	0.47	1.07	3.10	0.35	3.60
CNRM-CM5	3.56	2.08	4.52	2.17	1.13	0.59	1.71	1.08	0.49	1.57	2.12	4.24	0.50	0.98	2.64	0.37	3.16
CSIRO-Mk3.6.0	3.30	1.78	4.51	2.53	1.12	0.74	1.85	1.02	0.56	1.57	2.70	4.55	0.59	1.33	2.73	0.49	2.96
GFDL-CM3	3.60	1.95	4.79	2.45	1.12	0.72	1.84	1.03	0.49	1.52	2.72	4.83	0.56	1.37	2.87	0.48	3.20
HadGEM2-ES	3.26	2.50	5.44	2.17	0.75	0.55	1.30	0.76	0.44	1.20	2.50	4.84	0.52	1.15	2.70	0.43	4.32
INM-CM4	3.23	1.29	3.01	2.34	1.63	0.88	2.51	1.55	0.63	2.18	2.27	3.37	0.67	1.11	1.98	0.56	1.98
IPSL-CM5A-LR	3.43	2.04	5.21	2.56	0.99	0.69	1.68	0.88	0.50	1.38	2.88	5.57	0.52	1.24	3.22	0.39	3.46
IPSL-CM5A-MR	3.44	2.03	5.11	2.52	1.01	0.69	1.70	0.87	0.55	1.41	2.96	5.29	0.56	1.28	3.17	0.40	3.40
MIROC-ESM	4.59	2.16	5.56	2.58	1.32	0.80	2.13	1.10	0.61	1.71	3.22	5.27	0.61	1.37	3.03	0.45	3.47
MIROC5	4.47	1.51	3.74	2.48	2.11	0.85	2.96	1.74	0.71	2.46	2.73	3.74	0.73	1.32	2.10	0.63	2.12
MPI-ESM-LR	4.48	2.06	5.03	2.45	1.45	0.72	2.18	1.31	0.49	1.80	2.35	4.41	0.53	1.12	2.41	0.46	3.08
MPI-ESM-MR	4.52	2.04	4.81	2.36	1.54	0.68	2.22	1.39	0.52	1.92	2.36	4.38	0.54	0.99	2.55	0.39	2.94
MRI-CGCM3	3.55	1.56	4.03	2.59	1.62	0.66	2.28	1.37	0.44	1.81	1.91	3.66	0.52	0.82	2.08	0.40	2.19
NorESM1-M	3.78	1.39	3.61	2.61	1.79	0.94	2.73	1.50	0.67	2.17	2.66	3.76	0.71	1.24	2.18	0.57	2.11
mean	3.76	1.90	4.58	2.42	1.31	0.73	2.04	1.18	0.54	1.72	2.57	4.50	0.58	1.19	2.61	0.46	3.02
SD	0.50	0.35	0.72	0.15	0.35	0.11	0.43	0.27	0.08	0.33	0.32	0.66	0.08	0.17	0.40	0.08	0.65

## 2. Linearity and the step model

Linear systems theory assumes that the response of the system depends linearly on forcing, so that the response to a sum of forcings equals the sum of responses to individual forcings. Thus, if *X*_*i*_(*t*) is the response in some quantity of the system to forcing *F*_*i*_(*t*), the response to 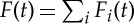 is 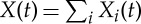. This implies that forcing *kF* gives response *kX* for any constant scaling factor *k*.

By applying this theory to the climate system, the ‘step model’ [18] estimates the response *X*(*t*) of any climate variable to a forcing scenario *F*(*t*) according to
2.1
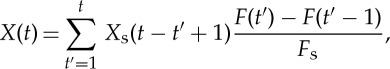
where the sum is over years, and *X*_s_(*t*) is the response after *t* years to a ‘step forcing’ *F*_s_, which is instantaneously switched on at *t*=0 and held constant indefinitely.

The step model thus regards the response to any time-dependent forcing scenario as the sum of responses to a succession of *t* annual forcing increments *F*(*t*)−*F*(*t*−1), with *F*(0)=0, and these individual responses are estimated by scaling the response to *F*_s_. The response to the step forcing is used rather like a Green function (which is the response to a *δ*-function forcing). The idea is illustrated schematically by Good *et al.* [19] (their fig. 1b), who show that it works well for global mean surface air temperature change and ocean heat uptake simulated in CMIP5 1pctCO2 and RCP scenarios, using the step response from the CMIP5 abrupt4xCO2 experiments, in which the CO_2_ concentration is quadrupled at *t*=0 and subsequently held constant, i.e. *F*_s_=*F*_4×_, the forcing due to 4×CO_2_.

Linearity as defined above and by Good *et al.* [20] is different from the property that the quantities of the system vary together in linear relationships as a function of state. Long & Collins [17] call that property ‘linearity in time’, because change in the state of the system is usually a matter of time. If the system is not linear, the *X*–*Y* relationship will not generally be linear, because *X* and *Y* will depend in different nonlinear ways on the forcing. If the system is linear, the step model will predict any quantities *X* and *Y* correctly and individually, even if they are not linearly related in time, but the form of the relationship between them will be a function of the time dependence of the system. For example, the increase in ocean heat content 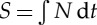, so the relationship in time between *N* and *S* is not generally linear; one possible time dependence of the system is *N*∝*t*⇒*S*∝*t*^2^∝*N*^2^. However, if the system is linear, meaning the step model predicts *N* correctly, it must also predict *S* correctly, regardless of the nonlinearity in time.

## 3. Two-layer model

The two-layer model [5,12,21–23] consists of the upper and the deep ocean, like the zero-layer model (§1), but the layers have finite non-zero heat capacities *C*_u_ and *C*_d_, *C*_u_≪*C*_d_. Their temperatures are *T*_u_ and *T*_d_ relative to initial equilibrium. The upper-layer temperature change *T*_u_ is identified with the global mean surface air temperature change *T*. The upper ocean participates in the surface energy budget and experiences the forcing, while the deep ocean receives a heat flux *H*=*γ*(*T*_u_−*T*_d_) from the upper ocean, with *γ* (W m^−2^ K^−1^) being a constant. Thus

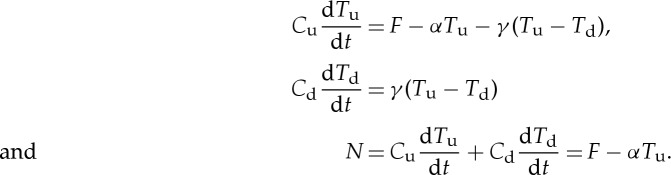
Geoffroy *et al.* [22] fitted the two-layer model to CMIP5 results for abrupt4xCO2. It is useful to consider how the model behaves qualitatively under this scenario, which clearly separates the various time scales of response to forcing, because it is used by the step model to predict the response to other scenarios. If a constant positive *F* is imposed at *t*=0, as in abrupt4xCO2, *T*_d_≪*T*_u_ while *t* is small, because the deep ocean has much larger heat capacity and warms up more slowly than the upper ocean. During this phase, *T*_d_ can be neglected in *γ*(*T*_u_−*T*_d_), and
3.1
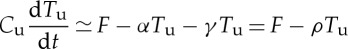
if we identify *κ* with *γ*, so that *ρ*=*α*+*γ*. Geoffroy *et al.* [22] call this form the ‘one-layer model’ and we will refer to it as the ‘upper-layer model’. In this approximation, the upper layer has finite and the deep layer infinite heat capacity. The solution of equation (3.1) is *T*_u_=(*F*/*ρ*)(1−*e*^−*ρt*/*C*_u_^). At *t*=0, *T*_u_=0 and d*T*_u_/d*t*=*F*/*C*_u_, but, as *T*_u_ rises, 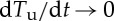, on the time scale *C*_u_/*ρ* characteristic of the upper ocean, which is 3.9 years using the CMIP5 ensemble mean parameters of Geoffroy *et al.* (tables 3 and 4 of [22]).

After 10–20 years, d*T*_u_/d*t*≪*F*/*C*_u_, and the upper-ocean thermal inertia can thereafter be neglected, because it stores only a small fraction of the heat uptake, which goes mainly into the deep ocean. By setting *C*_u_=0 in equation (3.1), we obtain the zero-layer model *F*=*ρT*_u_, which is valid while *T*_d_≃0⇒*N*=*H*=*γT*_u_ (or *N*=*κT*_u_ if we identify *κ* with *γ*).

For larger *t*, the zero-layer model is inaccurate because the deep ocean warms up, i.e. *T*_d_>0. However, *C*_u_ remains negligible, so
3.2
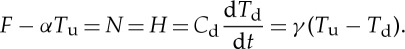
In this approximation, which we call the ‘deep-layer model’, the upper layer has zero and the deep layer finite heat capacity. The solution of equation (3.2) is

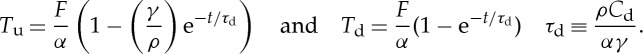
The formula given by Geoffroy *et al.* [22] for the ‘slow’ time scale of warming is approximated by our *τ*_d_ if *C*_d_≫*C*_u_. Using their CMIP5 ensemble-mean parameters, *τ*_d_=239 years.

**Figure 1.**
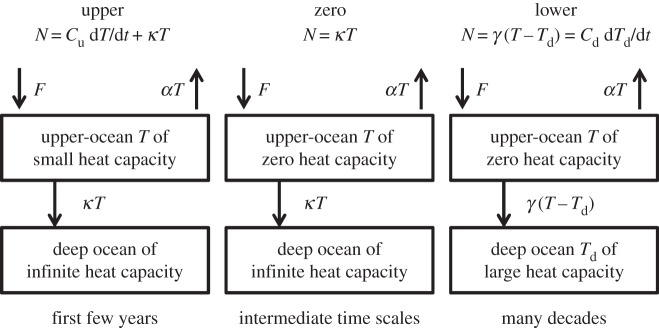
Schematic of the upper-, zero- and deep-layer models of ocean heat uptake, which are three approximations to the two-layer model, applicable on the time scales shown in the bottom row. In all versions of the model, the global energy balance is *F*=*N*+*αT*. Global mean surface air temperature change *T* is identified with the upper-layer temperature change.

**Figure 2.**
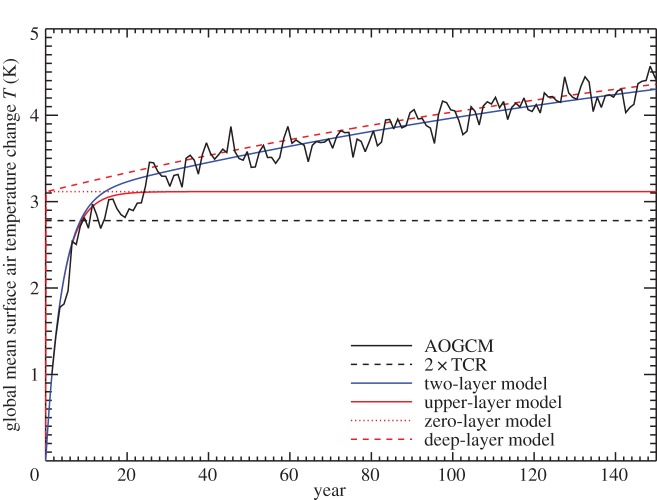
Global mean surface air temperature change *T* in the NorESM1-M AOGCM abrupt4xCO2 simulation compared with predictions by the zero-, one- and two-layer models.

The upper-, zero- and deep-layer approximations to the two-layer model are shown schematically in figure 1. As an example, we compare *T* simulated by the NorESM1-M AOGCM for abrupt4xCO2 with the prediction of the two-layer model fitted to this AOGCM by Geoffroy *et al.* [22] (figure 2, also shown in their fig. 2). The two-layer model is close to the forced response of *T* in the AOGCM, although slightly overestimating in the first 20 years. It does not generate unforced variability.

The upper-layer model prediction of *T* is indistinguishable from the two-layer one for about the first 10 years, but then levels off at *F*_4×_/*ρ*=3.1 *K*, consistent with equation (3.1). The zero-layer model predicts constant *T* at this same level from *t*=0. These two models cannot predict the continuing slow rise of *T* due to the warming of the deep ocean, which reduces *H*. Their *T* at long time scales would be twice the TCR (=*F*_2×_/*ρ*) if the two-layer model were exact.

Like the zero-layer model, the deep-layer model predicts *T*=*F*_4×_/*ρ* at *t*=0, because *C*_u_=0⇒*F*=*ρT*_u_ when *T*_d_=0. Unlike in the zero-layer model, *T*_d_ can rise in the deep-layer model, and *T*_u_ comes quite close to the AOGCM and two-layer model after about 20 years. Because the upper layer takes up heat in the initial phase of the two-layer model, but has no heat capacity in the deep-layer model, *T*_d_ is greater at all times in the latter. At large *t*, when *C*_u_ d*T*_u_/d*t* is negligible in both models, 0=*F*−*αT*_u_−*γ*(*T*_u_−*T*_d_)⇒*T*_u_=(*F*+*γT*_d_)/*ρ*. The greater *T*_d_ in the deep-layer model thus accounts for its small overestimate of *T*_u_ at large *t*. As 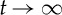, 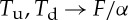 and 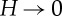 in both deep- and two-layer models.

**Figure 3.**
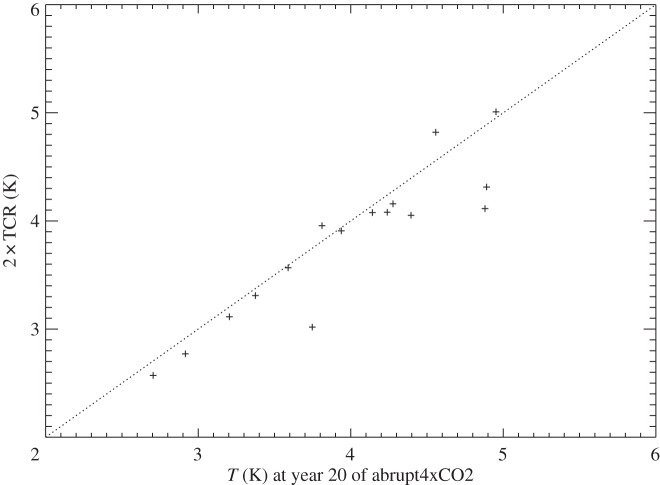
Comparison of the TCR with the global mean surface air temperature change *T* after 20 years of abrupt4xCO2 in the ensemble of 16 CMIP5 AOGCMs of table 1. The dotted line is 2×TCR=*T*.

In summary, the upper-layer model is accurate for about 20 years under abrupt4xCO2, and the deep-layer model thereafter. The zero-layer model is obviously inadequate because it predicts constant *T*. However, its prediction is quite accurate around *t*=20 years, when *F*=*ρT* is a good approximation, as can be seen for the example of NorESM1-M (figure 2), in which the zero-layer *T* is within 0.5 K of the two-layer model and the AOGCM for an intermediate phase spanning roughly years 10–60. In the set of 16 CMIP5 AOGCMs of table 1, the correlation between 2×TCR and *T* at year 20 in abrupt4xCO2 is 0.91 (figure 3). On average, the former is 5% smaller. The differences have the same causes as the inconstancy of TCRP under 1pctCO2, which we address in the following sections.

## 4. Climate response to increasing CO_2_

The inconstancy of the TCRP under increasing CO_2_ can be conveniently quantified by the ratio *T*_140_÷TCR, where *T*_140_=*T*(*t*=140 years), at the time of 4×CO_2_. This ratio would be 2 if TCRP were constant. In our ensemble of 16 CMIP5 AOGCMs, the ratio lies in the range 2.1–2.6 and has a mean of 2.4 (table 1), i.e. 20% larger than expected from constant TCRP.

**Figure 4.**
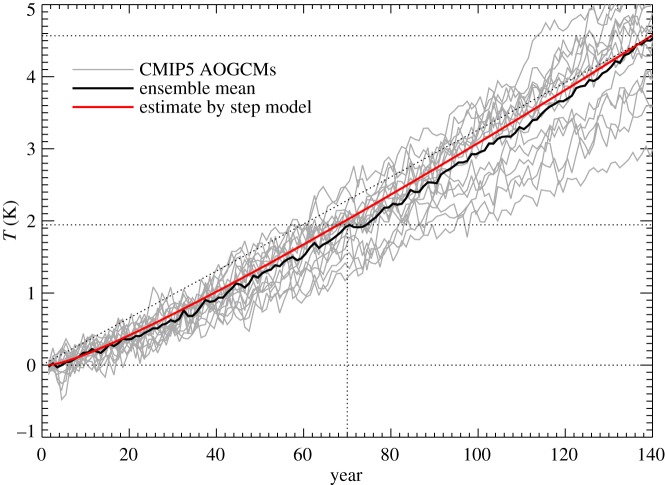
Global mean surface air temperature change *T* in CMIP5 1pctCO2 experiments, their ensemble mean and the ensemble mean of simulations by the step model using the abrupt4xCO2 response of each AOGCM. The ensemble-mean warming caused by the first and second doublings of CO_2_ can be read from the graph using the horizontal and vertical dotted lines. The diagonal dotted line indicates a constant d*T*/d*t*.

A constant geometric growth rate *p* of CO_2_ concentration, following *C*=*C*_0_*p*^*t*^, with *p*=1.01 for 1pctCO2, gives 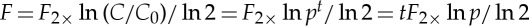, i.e. *F*∝*t* and 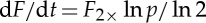 is a constant. Constant TCRP therefore predicts *T*=*F*/*ρ*∝*t* under 1pctCO2, but in the AOGCMs *T* rises at an accelerating rate (figure 4; [17]).

**Figure 5.**
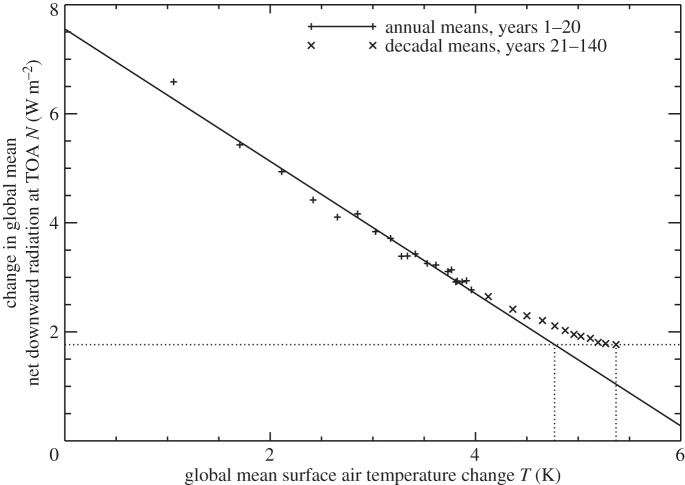
Relationship between annual means of the change in global mean net downward radiative flux *N* at the top of the atmosphere and the change in surface air temperature *T* in the ensemble mean of CMIP5 abrupt4xCO2 experiments. The solid line is a linear regression of *N* against *T* for years 1–20. The dotted lines indicate the time-mean *N* and *T* for years 131–140 and the estimate of *T* from the linear regression.

Under any constant forcing, *N*=*F*−*αT* is a straight line if *α* is constant [24]. In most CMIP5 abrupt4xCO2 experiments, the *N*–*T* relationship is not exactly linear [25]; there is a weak concave-upward curvature (figure 5) indicating a decrease in *α* [26–29]. Due to this effect, the ensemble-mean *T* after 140 years of constant 4×CO_2_ is about 10% larger than estimated from the *N* of that time using the linear regression of *N* against *T* from the first 20 years (as shown in figure 5 for the time-mean of years 131–140). The time-mean *F* during 140 years of 1pctCO2 is half of that in abrupt4xCO2, i.e. equal to 2×CO_2_ on average, and has less time to take effect because it rises gradually, so we might expect the effect of this nonlinearity on 1pctCO2 to be smaller, perhaps about 5%. Although it is in the right sense, and must contribute, it is too small to explain the inconstancy of TCRP by 20% on the model mean under 1pctCO2.

As a test case, we take the NorESM1-M AOGCM under 1pctCO2, for which *T*_140_/TCR is particularly large (table 1). Geoffroy *et al.* [22] fit the two-layer model to NorESM1-M assuming a linear *N*(*T*) relationship in abrupt4xCO2, while Geoffroy *et al.* [27] fit a modified version of the two-layer model, making *α* depend on *N* following the formulation of Held *et al.* [12] for the efficacy of ocean heat uptake, in order to reproduce the AOGCM nonlinear *N*(*T*) relationship. Their two versions of the two-layer model give indistinguishable results for NorESM1-M *T*(*t*) under 1pctCO2 (their fig. 4), and the same is true for the other AOGCMs they show, supporting our inference that the inconstancy of the climate feedback parameter under constant CO_2_ is not the reason for the inconstancy of the TCRP under increasing CO_2_.

**Figure 6.**
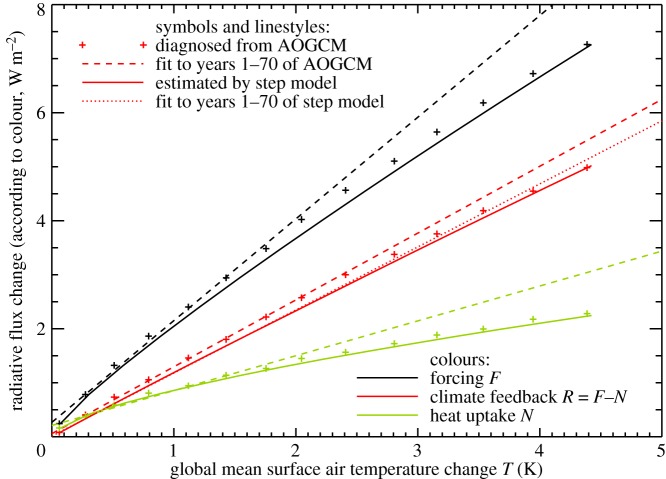
The relationship between the change in the energy budget of the climate system (radiative forcing *F*, climate feedback *R* and net downward radiative flux *N* at the top of the atmosphere, which equals net heat uptake) and the change in surface air temperature *T* in the ensemble mean of CMIP5 1pctCO2 experiments. The plus symbols show decadal means diagnosed from the AOGCMs. The dashed lines show linear functions of *T*, obtained by regression of the AOGCM results for years 1–70. The solid lines show estimates from the step model using *F*(*t*) for 1pctCO2 and the abrupt4xCO2 response. The dotted line shows a linear function of step-model *T*, obtained by regression of the step-model estimate of *R* for years 1–70.

The zero-layer model assumes that *F*=*ρT*. We calculate *F*(*t*) under 1pctCO2 from the logarithmic formula (see also §5) with *F*_2×_ in each AOGCM obtained by regressing *N* against *T* in its abrupt4xCO2 experiment [19,25], in which the *N*-intercept is *F*=*F*_4×_=2*F*_2×_ [24]. We avoid most of the nonlinearity of the abrupt4xCO2 *N*–*T* relationship by using only the first 20 years in the regression (figure 5), though even during these years there may be some curvature. In the CMIP5 ensemble mean of 1pctCO2 experiments, figure 6 shows that *F*∝*T* holds fairly well up to 2×CO_2_ (comparing the black plus symbols with the linear fit shown as a black dashed line), but it becomes inadequate thereafter as *ρ* decreases.

The same is true of the zero-layer model assumptions *R*=*F*−*N*=*αT* and *N*=*κT* with constant *α* and *κ* (figure 6, red and green plus symbols and dashed lines). In the AOGCMs, the climate feedback parameter *α* and the ocean heat uptake efficiency *κ* both tend to decrease (values for years 1–70 and years 121–140 are compared in table 1). This suggests that these two effects make distinct contributions to the inconstancy of the TCRP. We consider *α* in §5 and *κ* in §6.

## 5. Climate feedback

The curvature of *R*=*F*−*N* versus *T* under 1pctCO2, indicating declining climate feedback parameter *α*, is not reproduced by the step model, which predicts *R*∝*T*, i.e. constant *α* to a good approximation (figure 6, solid and dotted red lines), even though *α* is not constant in the abrupt4xCO2 experiment used by the step model (figure 5). This is a further demonstration that the decreasing *α* under constant 4×CO_2_ is not important for 1pctCO2.

**Figure 7.**
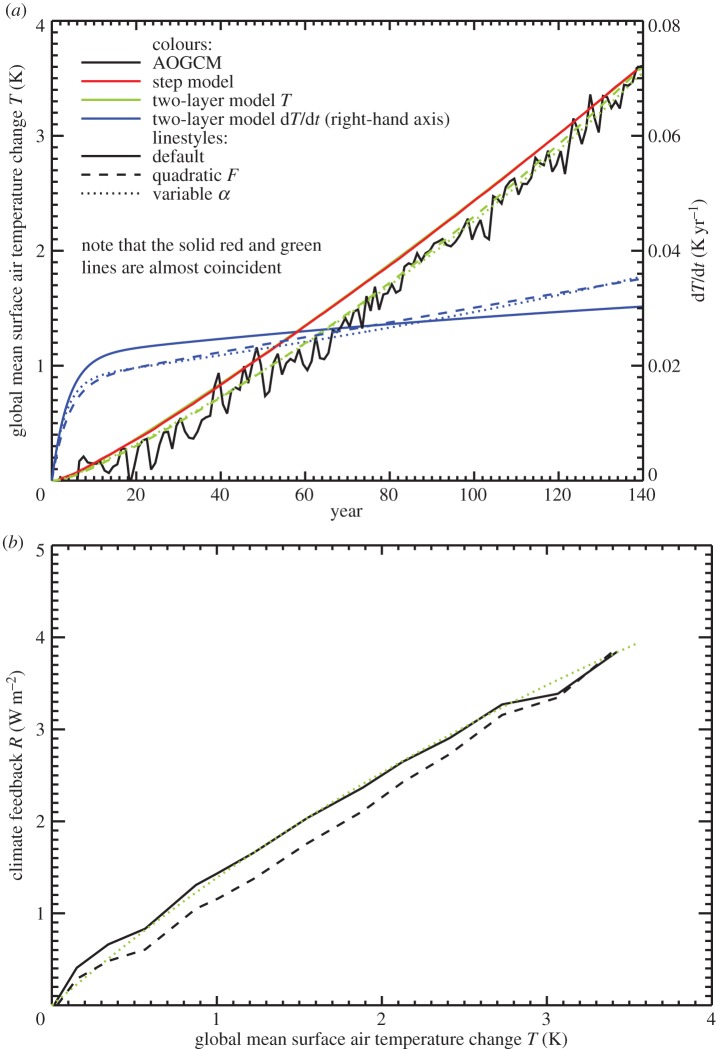
Response to 1pctCO2 in the NorESM1-M AOGCM and the step and two-layer models. (*a*) *T* (left-hand axis) and d*T*/d*t* (right-hand axis, two-layer model only) as a function of time *t*. (*b*) *R*=*F*−*N* versus *T*. The solid black AOGCM curve for *R* is calculated assuming the logarithmic formula for *F*.

The inaccuracy of the step model can also be seen as a function of time (figure 4, and fig. 2a of [19]). The error is largest in the middle decades of the 140-year experiment, and decreases subsequently. In the case of the NorESM1-M AOGCM, the step model gives particularly large errors in *T*(*t*) under 1pctCO2 (black and red lines in figure 7*a*). Using the parameters appropriate for NorESM1-M assuming a linear *N*(*T*) relationship for abrupt4xCO2 (tables 3 and 4 of [22]), we see that the two-layer model behaves very similarly to the step model (figure 7*a*, solid green and red lines, which are almost coincident). The inaccuracy of the step model means that the response to 1pctCO2 is not linear in the sense of linear systems theory (§2). We consider two possibilities to explain the nonlinear behaviour.

One possibility is that the logarithmic formula for *F*(CO_2_) is not accurate. The forcing caused by successive doublings of CO_2_ is shown to increase with CO_2_ in the GISS model E atmospheric general circulation model (AGCM) (table 1 of [30]), the BMRC AGCM [31] and the NCAR CCSM3 AOGCM (table 4 of [32]; fig. 2a of [33]). The HadCM3 AOGCM shows the opposite behaviour: 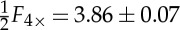 W m^−2^ is 10% less than *F*_2×_=4.28±0.16 W m^−2^.

In the results of Colman & McAvaney [31] (their fig. 1), the forcing caused by doubling CO_2_ concentration from *C* to 2*C* increases roughly linearly with 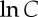 for 1–4×CO_2_. This suggests a modification of the usually assumed logarithmic dependence to

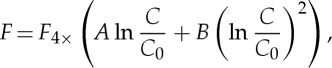
where *A* and *B* are constants, and *C*_0_ is the 1×CO_2_ concentration, for which *F*(*C*_0_)=0. Considering results of radiative transfer calculations from a line-by-line code, Byrne & Goldblatt [34] propose this same form as an approximate expression for CO_2_ forcing. With this form, 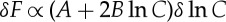, consistent with Colman and McAvaney. If *B*=0, the usual purely logarithmic formula 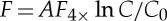 is recovered, where 
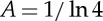
.

The quadratic formula can be fitted to *F*(*C*), requiring *F*(*C*_0_)=0, by linear regression of 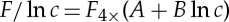 against 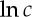, where *c*≡*C*/*C*_0_. The results of Hansen *et al.* [30] for *F*(*C*) with *c* in the range 1–16 are thus estimated with a RMS error of 0.12 W m^−2^ using *A*=0.66 and *B*=0.051, and those of Jonko *et al.* [32] for effective radiative forcing with *c*=2,4,8 with an error of 0.038 W m^−2^ using *A*=0.58 and *B*=0.109. The expression given by Byrne & Goldblatt [34] is equivalent to *F*_4×_=8.12 W m^−2^, *A*=0.66, *B*=0.048.

In the 1pctCO2 scenario, 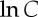 increases linearly with time, so *F*(*t*)=*F*_4×_(*A*′*t*+*B*′*t*^2^), with 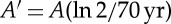 and 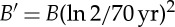. Using this quadratic form for *F* as a function of time with *F*_4×_=6.2 W m^−2^ for NorESM1-M [22,25] largely eliminates the *T* error in the two-layer model (the dashed green line in figure 7*a* follows the trajectory of the black line). This is simply because the quadratic *F* initially increases more slowly than the linear *F*, and later more quickly, and *T* behaves likewise (compare the dashed and solid blue lines in figure 7*a*). Using the quadratic *F* to compute *R*=*F*−*N* with the AOGCM *N* and *T* straightens the *R*–*T* relationship in the AOGCM (compare the solid and dashed black lines in figure 7*b*). This explanation of the error thus allows *α* to be constant; its apparent inconstancy in figure 6 would be due to the incorrect estimate of *F*(*t*).

The second possibility to explain the *T* error is that *α* depends on time or on *C*—these two possibilities cannot be distinguished in 1pctCO2, since *F*∝*t*. Jonko *et al.* [32] find that *α* decreases for successive doublings of CO_2_ in CCSM3. In the HadCM3 AOGCM, *α* is about 20% larger for constant 2×CO_2_ than 4×CO_2_ (1.47±0.10 and 1.25±0.04 W m^−2^ K^−1^, respectively, from ensemble means of seven integrations). Using the quadratic formula for *F*(*C*) fitted for HadCM3 with *c*=*C*/*C*_0_=2 and 4, we can calculate *α*=(*F*−*N*)/*T* during the 1pctCO2 experiment. We find that it declines linearly in time (not shown). Regressing *α*(*t*) against *t*, we estimate that *α*=1.5 W m^−2^ K^−1^ and 1.2 W m^−2^ K^−1^ at *t*=70 and 140 years, when *c*=2 or 4, respectively, in agreement with the results from abrupt2xCO2 and abrupt4xCO2. Extrapolating backward to the start of the experiment indicates *α*=1.8 W m^−2^ K^−1^ for *c*=1. Thus, *α* declines by about 40% during the 140 years of 1pctCO2 in HadCM3.

For the sake of illustration, we therefore assume likewise in the two-layer model for NorESM1-M that *α* for infinitesimal CO_2_ perturbations with respect to the control CO_2_ is 40% larger than *α* for 4×CO_2_ (1.11 W m^−2^ K^−1^), and that *α* decreases linearly with time during the 1pctCO2 scenario. With this form for *α* (using *F* linear in time, as we did originally), we again largely eliminate the error in *T* (compare the dotted green and solid black lines in figure 7*a*). The explanation is somewhat different from that for the quadratic *F*. Initially, *T*=0, so *N*=*F* for both constant and decreasing *α* and *T* rises initially at the same rate (solid and dotted blue lines in figure 7*b*). As soon as *T*>0, the larger *α* reduces *N*=*F*−*αT*, so the rate of warming with variable *α* falls behind. Later on, *α* becomes more similar in the two cases, but the case with variable *α* by then has lower *T*, so *N* is larger, the rate of warming becomes greater than for constant *α*, and the error in *T* decreases. Using this form for *α*, the two-layer model reproduces the nonlinear *R*(*T*) relationship of the AOGCM (dotted green and solid black lines in figure 7*b*).

Thus, the error in the estimate of *T* for NorESM1-M under 1pctCO2 by the step and two-layer models can be explained equally well by *F* increasing more rapidly than logarithmic with CO_2_, or by *α* decreasing during the experiment (as a function of CO_2_ concentration or of time). These are nonlinear effects in the sense of §2. (They were mitigated by Good *et al.* [19] in their use of the step model to predict differences in *T* between RCPs, rather than predicting *T* in individual RCPs.) Either effect could account for the deviation of the step model from the AOGCM in intermediate years of 1pctCO2 in the CMIP5 ensemble (figure 4). Consequently either could also explain the apparent inconstancy of the climate feedback parameter (figure 6). The two are distinct physical explanations, but with the experiments currently available we cannot tell which of them is more important in explaining the CMIP5 behaviour. (They could be distinguished using abrupt2xCO2 experiments; see §8.)

## 6. Ocean heat uptake

The curvature of the *N*–*T* relationship under 1pctCO2 indicates declining ocean heat uptake efficiency *κ*=*N*/*T* [35]. This behaviour is well reproduced by the step model (figure 6, solid and dotted green lines). It follows that the behaviour of ocean heat uptake efficiency under 1pctCO2 must be entirely a consequence of effects that are also observed under abrupt4xCO2.

**Figure 8.**
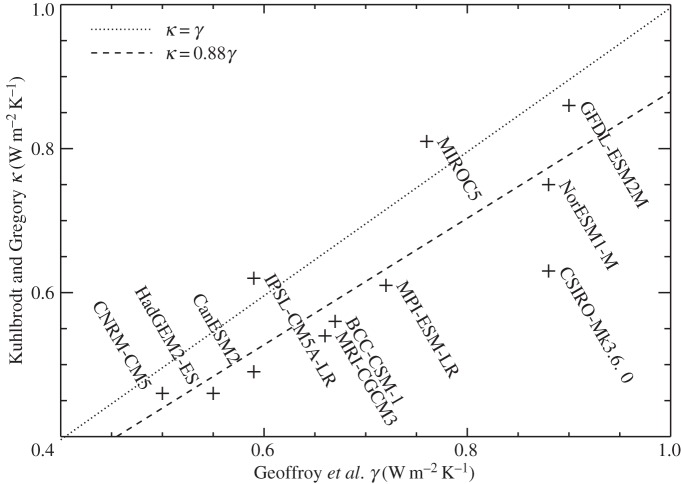
Relationship between the ocean heat uptake efficiency *κ* diagnosed from CMIP5 AOGCMs [11] and the thermal coupling *γ* in the two-layer model fitted to AOGCMs [22].

Therefore, the ocean heat uptake efficiency for 1pctCO2 should be related to the zero-layer approximation of the two-layer model, which applies on the time scale of a few decades, as we showed above (§3). This can be tested for CMIP5 models by comparing the values of *κ* obtained from regression of *N*(*t*) against *T*(*t*) in 1pctCO2 experiments [11] for years 1–70, during which constant *κ* is a reasonably good approximation (figure 6), with those obtained for *γ* by fitting the two-layer model to abrupt4xCO2 experiments [22]. These two datasets and fitting procedures are entirely independent, and there is a highly significant correlation of 0.83 between *κ* and *γ* in the set of 11 AOGCMs for which both are available (figure 8).

Because *T*_d_≠0, the zero-layer approximation of *N*=*H*=*γ*(*T*_u_−*T*_d_) by *κT*_u_ requires *κ*=*N*/*T*_u_=*γ*(1−*T*_d_/*T*_u_)<*γ*, as can be seen by comparison with the 1 : 1 line in figure 8. The correlation of *κ* with *C*_d_ of Geoffroy *et al.* [22] (their *C*_0_) is 0.63, which is significant at the 5% level. We suggest that this is because, for a given *T*_u_ and *N*, a larger *C*_d_ gives a smaller *T*_d_ and implies a larger *κ*. There is no significant correlation of *κ* with *C*_u_. We conclude that the spread of ocean heat uptake efficiency across models is predominantly caused by the spread in the strength of the thermal coupling between upper and deep oceans.

The ‘cold start’ effect [36,37] is a consequence of the inconstancy of *κ*. It is the observation that under steadily increasing forcing, beginning from a steady state, the initial rate of warming is less than the rate after a few decades. A qualitative explanation of the effect can be given in terms of the zero-layer model *T*=*F*/*ρ*, in which the rate of warming d*T*/d*t*=(1/*ρ*) d*F*/d*t*, assuming the rate of change of *ρ* is relatively small. If *κ* declines, so does *ρ*, and hence d*T*/d*t* increases with time under a forcing increasing at a constant rate d*F*/d*t*=*S*, as in 1pctCO2.

**Figure 9.**
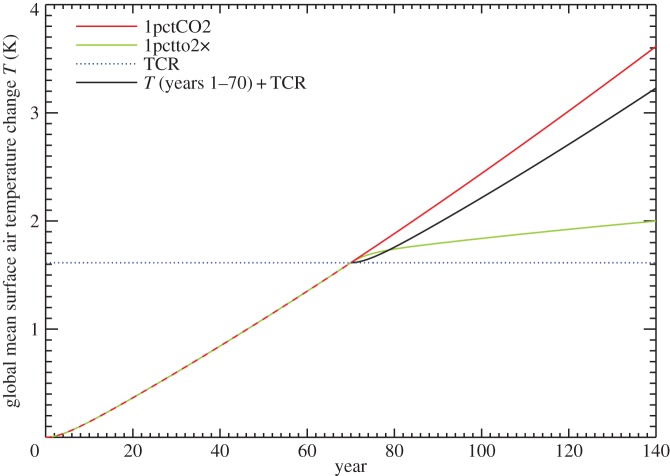
Comparison of global mean surface air temperature change *T* under the 1pctCO2 and 1pctto2x scenarios in the two-layer model fitted to the NorESM1-M AOGCM. The two scenarios are the same for years 1–70. The black line is *T* in years 1–70 translated rightward by 70 years and upward by the TCR, i.e. *T* at year 70.

This phenomenon is illustrated using results from the two-layer model (fitted to NorESM1-M, [22]) in figure 9, in which we compare *T*(*t*) during the first and second doublings of CO_2_ (black and red lines). The cold start effect is the rapid separation of the lines in the first decade, but they continue to diverge thereafter as *κ* declines. Since the two-layer model is linear in its response to forcings, *T* for years 71–140 of 1pctCO2 (red line in figure 9, CO_2_ increasing from 2×CO_2_ to 4×CO_2_, following CO_2_ increasing at 1% yr^−1^ for 70 years) is the sum of *T* for years 1–70 of 1pctCO2 (CO_2_ increasing at 1% yr^−1^ up to 2×CO_2_, the difference between the black and blue lines) and *T* for years 71–140 of the 1pctto2x scenario (constant 2×CO_2_, following CO_2_ increasing at 1% yr^−1^ for 70 years, green line). This decomposition suggests the interpretation that the *T* increase for the second doubling of CO_2_ is greater than the TCR because of the ‘commitment’ to further warming accumulated during years 1–70 and realized during years 71–140 of 1pctto2x, during which *T* rises (green line) while *F* is constant at *F*_2×_ and *N*=*F*−*αT* is declining. This last statement implies that ocean heat uptake efficiency *κ*=*N*/*T* is decreasing. Thus, the interpretations of this paragraph and the preceding one are equivalent.

**Figure 10.**
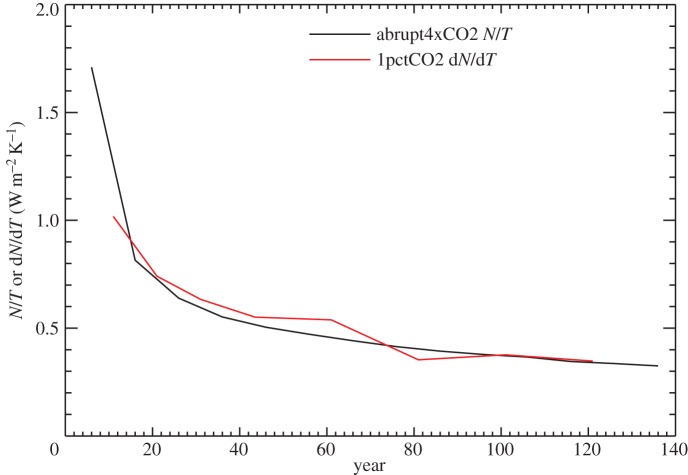
Comparison of d*N*/d*T* as a function of time in 1pctCO2 with *N*/*T* as a function of time in abrupt4xCO2 for the CMIP5 ensemble mean.

Under abrupt4xCO2, *N* decreases from *F* at *t*=0 to zero in the final steady state, while *T* increases from 0 to *F*/*α* (figure 5, noting that *α* is not constant, as discussed in the previous section). Hence, *κ*=*N*/*T* declines continuously in abrupt4xCO2, from infinity to zero (figure 10). Given this behaviour, it is perhaps surprising that *κ* can be treated as constant for a few decades under 1pctCO2. A quantitative understanding of this can be gained from the step model.

The step response and the scenario response are in a special relationship in the case of constant d*F*/d*t*, because *F*(*t*)−*F*(*t*−1)=*S* for all *t*, and equation (2.1) becomes

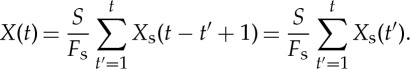
Although we do not apply the step model in a continuous-time form, it helps the interpretation to regard this as

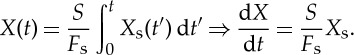
Thus, the *rate of change* of the 1pctCO2 response after *t* years is proportional to the *response itself* to the abrupt4xCO2 step after *t* years.

Hence
6.1
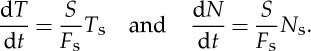
Under abrupt4xCO2, *T*_s_ is initially zero and rapidly increases, but after the upper ocean time scale of 10–20 years it tends to level off, with comparatively slow warming thereafter (figures 2 and 5). Therefore, under 1pctCO2, the *rate* of warming d*T*/d*t* is initially zero but rapidly increases on the upper ocean time scale. This is the cold start effect. Thereafter, d*T*/*dt* increases slowly under 1pctCO2 because *T*_s_ increases slowly in response to step forcing.

Moreover,
6.2
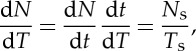
i.e. the tangent slope of the *N*(*T*) relationship in 1pctCO2 equals the slope of the line from the origin to (*T*_s_,*N*_s_) after the same time in abrupt4xCO2 (figure 10). These quantities are initially large and decrease as time passes. Therefore, *N* under 1pctCO2 rises steeply with *T* at first (large d*N*/d*T*), but at a continuously decreasing rate (d*N*/d*T* decreases, solid green line in figure 6). This slow decline in d*N*/d*T* and hence in ocean heat uptake efficiency, which contributes to the increase in TCRP under 1pctCO2, arises from the warming of the deep ocean on multi-decadal time scales.

## 7. Transient climate response in representative concentration pathways

Forster *et al.* [38] find in CMIP5 experiments that *ρ* for 2000–2050 is smaller (i.e. TCRP is greater) for RCP8.5 than for 1pctCO2, and smaller for RCP4.5 than for RCP8.5. The forcing in RCPs is dominated by greenhouse gases, especially CO_2_, throughout the twenty-first century, and increasingly so as time passes and anthropogenic aerosol forcing becomes relatively smaller. From our findings in §5, we might suppose that the *ρ* dependence on scenario could partly be due to *α* decreasing with increasing CO_2_. However, the forcing used by Forster *et al.* to calculate *ρ* was estimated using the method of Forster & Taylor [39], which assumes a constant *α*, according to *F*=*N*+*αT*⇒*ρ*=*F*/*T*=*N*/*T*+*α*. Hence, the *ρ* variation they find can only come from ocean heat uptake efficiency, although if *F* were re-evaluated in these scenarios using prescribed sea-surface conditions [12,30,40] variation might be revealed in *α* as well.

The average rate of forcing increase from 2000 to 2050 is equivalent to 1.1% yr^−1^ of CO_2_ concentration in RCP8.5 and 0.7% yr^−1^ in RCP4.5 (using data from [2]), both different from 1pctCO2. However, from equation (6.1), with *T*(0)=0 and *N*(0)=0, we obtain

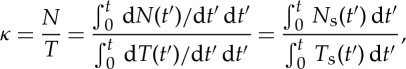
which does not depend on *S*=d*F*/d*t*. Therefore, *κ* will be the same, at a given time, under a scenario of constant forcing increase at any constant rate *S*, implying that the differences in the average rate of forcing increase in 1pctCO2 and RCPs do not explain the *ρ* variation noted by Forster *et al.*

Instead, we suggest that the explanation has two aspects. First, the value of *ρ* for 1pctCO2 corresponds to the TCR, i.e. year 70, the time of 2×CO_2_, whereas by 2050 in the RCP experiments, which are preceded by historical simulations, the forcing has already been increasing for a couple of centuries. Hence, the *κ* and *ρ* for the RCPs are smaller than for the TCR because of the continuous decline of ocean heat uptake efficiency as time passes. Second, under RCP4.5, although the forcing continuously increases (d*F*/d*t*>0) during the twenty-first century, it tends to stabilize (d^2^*F*/d*t*^2^<0). Referring to equation (2.1), this means that *F*(*t*′)−*F*(*t*′−1) is greater for smaller *t*′, giving greater weight to *X*_s_ for later times, when *N*_s_/*T*_s_ is smaller. Therefore, d*N*/d*T* and hence *κ* and *ρ* decrease more rapidly in stabilization scenarios such as RCP4.5 than in scenarios such as RCP8.5 having constant or increasing d*F*/d*t*.

The decline in ocean heat uptake efficiency is one of the reasons why TCRP increases, as discussed in §§1 and 6. The zero-layer model suggests that projections of *T* can be made by scaling the TCR=*F*_2×_/*ρ*, according to *T*=*F*/*F*_2×_×TCR=*F*/*ρ*=*F*/(*α*+*κ*). If *κ* declines, this formula will give an underestimate of *T*. Specifically, using *κ*_2×_ for the time of 2×CO_2_ under 1pctCO2, i.e. as in the TCR, instead of the *κ*_RCP_ actually prevailing at the time in the RCP projection will lead to a fractional error in *T* of

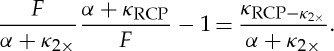
In our set of 16 CMIP5 AOGCMs, the ensemble-mean *κ*_RCP_ for the time-mean of 2081–2100 is 0.58 W m^−2^ K^−1^ under RCP8.5 and 0.46 W m^−2^ K^−1^ under RCP4.5, while *κ*_2×_=0.73 W m^−2^ K^−1^ and *α*=1.31 W m^−2^ K^−1^ (the latter assumed constant, table 1). Hence, scaling the TCR will underestimate *T* in 2081–2100 by 7% and 13% for RCP8.5 and RCP4.5, respectively, due to the decline in *κ*.

The estimated equilibrium warming *F*/*α* exceeds the zero-layer estimate *F*/(*α*+*κ*_RCP_) by 44% and 35% in 2081–2100. This shows the important influence of ocean heat uptake on the mean magnitude of *T*, which is the reason why the TCR gives a better indication of the expected *T* than the ECS=*F*_2×_/*α*.

On the other hand, the model spread in *T* under 1pctCO2 and RCPs is dominated by the spread in *F* and *α*, with *κ* being comparatively uninfluential [11,38,41]. Consequently, the correlation across the models of *T* for 2016–2035 under RCP8.5 and RCP4.5 with the TCR (0.69 and 0.68, respectively) is not much higher than with the ECS (0.65 for both RCPs). For 2081–2100, the correlation of *T* with TCR (0.79 and 0.76) is *lower* than with the ECS (0.83 and 0.80). This is because *κ* declines at different rates in the various models, in such a way that using *κ*_2×_ degrades the correlation. By contrast, the correlation of *T* with *F*_2×_/(*α*+*κ*_RCP_), i.e. using the actual *κ*_RCP_, is higher than with the ECS (0.84 for both RCPs).

However, the correlation of *T* for 2081–2100 under RCP8.5 and RCP4.5 with *T*_140_ (*T* at the time of 4×CO_2_ under 1pctCO2) is greater still (0.90 and 0.85). This suggests that the decline of *α* or the non-logarithmic increase in CO_2_ forcing (§5) might also affect the RCP projections, and therefore *T*_140_ might be a better model metric than the TCR for the magnitude of climate change projected for the end of the 21st century.

## 8. Discussion and conclusion

The 1pctCO2 experiment (with the CO_2_ concentration increasing at 1% yr^−1^) has been used since the early 1990s in AOGCMs to make an idealized projection of time-dependent climate change. The standard metric for model comparison of the magnitude of climate change is the TCR, defined as the global mean surface air temperature change *T* under this scenario at the time of 2×CO_2_ (after 70 years). In all CMIP5 AOGCMs, the global warming in 1pctCO2 under the second doubling, from 2×CO_2_ to 4×CO_2_, is greater than the TCR, by 40% in the model mean. We describe this as an increase in the TCRP, which we define as the increase in *T* per unit increase in forcing (in K W^−1^ m^2^, in any scenario).

The zero-layer model of the Earth energy budget assumes that *T* scales with *F*, according to *F*=*ρT*, where *F* is the effective radiative forcing and *ρ*=*α*+*κ* is a constant, the sum of the climate feedback parameter *α* and the ocean heat uptake efficiency *κ*. In this model, ocean heat uptake thus looks formally like a climate feedback; since *α* and *κ* are both positive, the warming surface climate balances the forcing by losing heat at an increasing rate to space (through *αT*) and into the deep ocean (through *κT*). In the zero-layer model, the TCRP is a constant 1/*ρ*. The inconstancy of TCRP implies that, although useful as an interpretative picture, the zero-layer model is not accurate. We find that the TCRP increases during the 1pctCO2 experiment because both *α*=(*F*−*N*)/*T* and *κ*=*N*/*T* decline, where *N* is the rate of heat uptake by the climate system, which is practically all stored in the ocean.

The abrupt4xCO2 experiment was newly introduced in CMIP5. It has proved useful in clarifying the concepts of forcing and feedback [42], and allows the 4×CO_2_ forcing to be evaluated by the regression method. Furthermore, the climate response to abrupt4xCO2 can be used to estimate the response to any forcing scenario, by regarding it as a succession of step changes in forcing, and assuming that the responses to the steps combine linearly (in the sense of systems theory) [18].

Using the step model, we have shown that the decline of ocean heat uptake efficiency *κ* during 1pctCO2 is a linear response. In the first couple of decades, ocean heat uptake efficiency is particularly large because the upper ocean is absorbing most of the heat, so that d*T*/d*t* is restrained by its heat capacity; this is the ‘cold start’ effect. Thereafter, *κ* continues to decline because the upper ocean loses heat into the deep ocean with decreasing efficiency as the deep ocean warms up. The thermal coupling between the upper and deep ocean, quantified by fitting the abrupt4xCO2 response using a two-layer model, is strongly correlated with the zero-layer *κ*. This means that the model spread in *κ* is quantitatively accounted for by the spread in this thermal coupling. As an approximation to the two-layer model, the zero-layer model is most accurate at intermediate times, after the initial rapid warming of the upper layer, but before the deep layer has warmed substantially. Consequently, the TCR can be estimated within about 5% from *T* after about 20 years in the abrupt4xCO2 scenario.

Explaining the spread in physical terms requires analysis of the processes of ocean vertical heat transport (advection, diffusion, etc.; e.g. [21,43]). Although the two-layer model is quite accurate and useful as an exploratory tool, it is of course a simplification; the behaviour of ocean heat uptake in three-dimensional ocean GCMs does not look like that of two discrete layers. Ocean heat uptake is often treated in simple climate models as vertical diffusion (e.g. [17,44,45]); this can reproduce AOGCM global mean heat uptake reasonably well on century time scales, but it too is not a good representation of the processes involved. Moreover, linearity is inaccurate in the ocean interior, especially in more complex scenarios [5].

In contrast to ocean heat uptake efficiency, the step model shows that the apparent decline of the climate feedback parameter *α* during 1pctCO2 is not a linear response. Using the two-layer model, we have shown that it could be explained by *α* decreasing as CO_2_ concentration increases, or by CO_2_ forcing rising more rapidly with CO_2_ concentration than the usually assumed logarithmic dependence. The physical reasons for these phenomena require analysis. Both of these possibilities have been demonstrated in some GCMs, but we cannot distinguish them with CMIP5 experiments. To meet this need, we recommend that abrupt2xCO2 (instantaneously doubled CO_2_) experiments should be carried out as well as abrupt4xCO2, as has been proposed for the CFMIP subproject of CMIP6.

To evaluate the forcings in CMIP5 historical and RCP experiments, Forster *et al.* [38] used the method of Forster & Taylor [39], which depends on the assumption that *α* is constant and the same for all forcing agents, and uses *F*=*N*+*αT* with *α* for CO_2_ to estimate forcings due to other agents given *N* and *T* from the AOGCM. If *α* declines with increasing CO_2_, this method will increasingly underestimate the AOGCM forcing. In general, if *α* cannot be assumed to be constant, it can be computed only if *F* has been separately diagnosed. This is the purpose of the proposal by the Radiative Forcing Model Intercomparison Project (RFMIP) subproject of CMIP6 to perform experiments using AGCMs with prescribed sea-surface conditions and various forcings. We expect that the results will yield important new information about the dependence of *α* on time, climate state and the nature of the forcing agent.

In the step-model estimate of 1pctCO2, the TCRP increases, but not as rapidly as it does in the AOGCMs, because the step model reproduces only the linear part of the effect, relating to ocean heat uptake, not the nonlinear part, relating to CO_2_ forcing or climate feedback. Consequently, under 1pctCO2 *T* warms initially more quickly in the step model than in the AOGCMs, but later on more slowly (figure 4). It is notable, however, that the step model converges on the AOGCM results at 4×CO_2_, after 140 years. This is because the step model is constructed from the AOGCM abrupt4xCO2 experiment, and hence uses the AOGCM *α* and *F* for 4×CO_2_, whatever the values that prevail in the AOGCM earlier in the 1pctCO2 experiment. Since the step model correctly reproduces the heat uptake *N* as a function of *T* throughout the experiment, the energy budget *F*=*N*+*αT* is satisfied at 4×CO_2_ in the step model as in the AOGCM.

Long & Collins [17] calculate the ECS *F*_2×_/*α* as a function of time in abrupt4xCO2 and 1pctCO2 (their fig. 2 shows MIROC-ESM as an example). (They correctly call this quantity the ‘effective climate sensitivity’, the distinction from ECS being that *α* is estimated from a non-equilibrium state, but the definition is otherwise the same; see [46].) They show that ECS increases during 1pctCO2. This is because of the same nonlinear effects that explain the inaccuracy of the step model and the apparent decline of *α*. ECS also increases slightly under 4×CO_2_. This is due to the tendency of *α* to decline under fixed CO_2_ (e.g. [25]), because of evolving patterns of sea-surface temperature or other effects leading to a nonlinear dependence of global mean radiative fluxes on global mean surface temperature change (e.g. [28,29,47]). This inconstancy of the climate feedback parameter under fixed CO_2_ must also contribute to the increase of TCRP under 1pctCO2, but it is much less important than the other effects we have described.

The pronounced inconstancy of TCRP limits the usefulness of the TCR as a model metric, though it remains relevant as an indicator of the approximate magnitude of *T* in response to future anthropogenic forcing. Assuming that AOGCMs are qualitatively realistic, we expect that the TCRP of the real world also increases during time-dependent climate change forced by increasing CO_2_. Therefore, the TCRP inferred from observed climate change could be smaller than that applicable to future climate change [48]; this could be part of the reason for observational estimates of TCR being lower than model-based ones [2]. Furthermore, scaling the TCR by *F*/*F*_2×_ will underestimate future *T* in response to higher CO_2_ [4], though the error would be partly alleviated by using the correct dependence of *F* on CO_2_, if it is not accurately logarithmic as usually assumed. The underestimate will be more serious for stabilization scenarios, in which *κ* declines more quickly. The decline of *κ* at different rates in different models means that *T* in the late twenty-first century under RCP4.5 and RCP8.5 correlates less strongly with the TCR than with the ECS (which does not depend on *κ*), and less strongly with the ECS than with *T*_140_ (*T* at the time of 4×CO_2_, at year 140, under 1pctCO2). Hence, *T*_140_ might be a better metric for model intercomparison than the TCR or the ECS for the expected magnitude of *T* in the late twenty-first century.

## Data accessibility

The CMIP5 data analysed in this work are available through http://pcmdi9.llnl.gov/esgf-web-fe.
